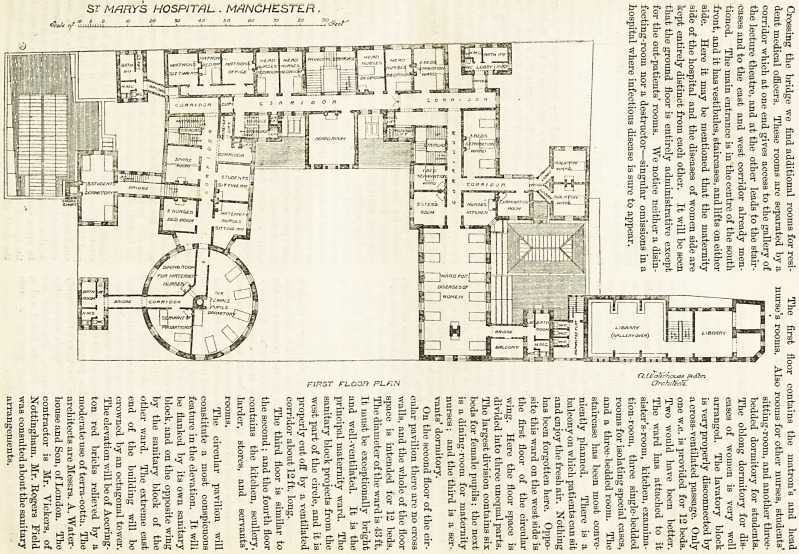# Hospital Construction

**Published:** 1899-12-02

**Authors:** 


### THE INSTITUTIONAL WORKSHOP.

### HOSPITAL CONSTRUCTION.

### ST. MARY'S HOSPITAL, MANCHESTER.

This hospital is intended for maternity cases and for diseases of women. The south front faces Gloucester Street, and the north front adjoins the towing path of the canal. The chief elevation, including the circular dormitory, faces due south, and will obtain the greatest possible amount of sunshine, while the oblong dormitories have their ends facing south, and the sides of the wards will get both morning and afternoon sun.

At first sight the ground plan has a somewhat confused appearance, but on closer observation it is seen to include many good points, and it must be said that the available space has been carefully utilised. On the east lie the out-patients' waiting-rooms, three in number, and four physicians' rooms. The waiting-rooms communicate with each other, and the patients enter at one end and leave by the other, thus avoiding confusion. Immediately behind the central waiting-room is the museum, and joining it is the dispensary, having a hatch for the delivery of medicine into the large waiting-room. Further north are two bedrooms for resident medical officers. Here a corridor runs east and west. On the north side are the lavatories, carefully cut off from the main corridor by a ventilating passage. Then come more rooms for the resident medical officers, secretary's room, principal staircase, and chapel. Opening from a roadway, or open court, are the mortuary and post-mortem room, and at the extreme west end is the laundry. Adjoining this, but approached by a bridge from the main building, is a three-bedded dormitory for students.

Crossing the bridge we find additional rooms for resident medical officers. These rooms are separated by a corridor which at one end gives access to the gallery of the lecture theatre, and at the other leads to the staircases and to the east and west corridor already mentioned. The main entrance is in the centre of the south front, and it has vestibules, staircases, and lifts on either side. Here it may be mentioned that the maternity side of the hospital and the diseases of women side are kept entirely distinct from each other. It will be seen that the ground floor is entirely administrative except for the out-patients' rooms. We notice neither a disinfecting-room nor a destructor—singular omissions in a hospital where infectious disease is sure to appear.

The first floor contains the matron's and head nurse's rooms. Also rooms for other nurses, students' sitting-room, and another three-bedded dormitory for students. The oblong dormitory for diseases of women is very well arranged. The lavatory block is very properly disconnected by a cross-ventilated passage. Only one w.c. is provided for 12 beds. Two would have been better. The ward has attached to it sister's-room, kitchen, examination-room, three single-bedded rooms for isolating special cases, and a three-bedded room. The staircase has been most conveniently planned. There is a balcony on which patients can sit and enjoy the fresh air. Nothing has been forgotten here. Opposite this ward on the west side is the first floor of the circular wing. Here the floor space is divided into three unequal parts. The largest division contains six beds for female pupils; the next is a dining-room for maternity nurses; and the third is a servants' dormitory.

On the second floor of the circular pavilion there are no cross walls, and the whole of the floor space is intended for 12 beds. The diameter of the ward is 43 ft. It must be exceptionally bright and well-ventilated. It is the principal maternity ward. The sanitary block projects from the west part of the circle, and it is properly cut off by a ventilated corridor about 12 ft. long.

The third floor is similar to the second; and the fourth floor contains the kitchen, scullery, larder, stores, and servants' rooms.

The circular pavilion will constitute a most conspicuous feature in the elevation. It will be flanked by its own sanitary block, and on the opposite wing by the sanitary block of the other ward. The extreme east end of the building will be crowned by an octagonal tower. The elevation will be of Accrington red bricks relieved by a moderate use of terra-cotta. The architects are Messrs. A. Waterhouse and Son, of London. The contractor is Mr. Vickers, of Nottingham. Mr. Rogers Field was consulted about the sanitary arrangements.

## Figures and Tables

**Figure f1:**
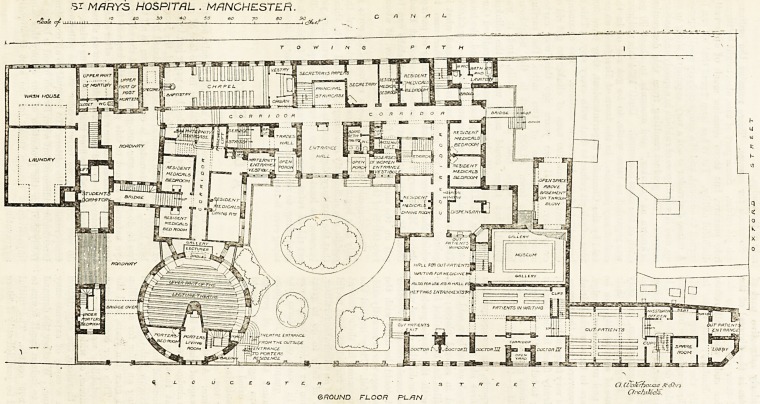


**Figure f2:**